# Digital twins suggest a mechanistic basis for differing responses to increased flow rates during high-flow nasal cannula therapy

**DOI:** 10.1186/s40635-025-00773-5

**Published:** 2025-06-26

**Authors:** Hossein Shamohammadi, Sina Saffaran, Roberto Tonelli, Valentina Chiavieri, Giacomo Grasselli, Enrico Clini, Tommaso Mauri, Declan G. Bates

**Affiliations:** 1 School of Engineering, University of Warwick, Coventry, CV4 7AL UK; 2 Respiratory Diseases Unit, Department of Medical and Surgical Sciences, University Hospital of Modena, University of Modena and Reggio Emilia, Modena, Italy; 3 Department of Anesthesia, Critical Care and Emergency, Fondazione IRCCS Ca’ Granda, Ospedale Maggiore Policlinico, Via F. Sforza 35, 20122 Milan, Italy; 4 Department of Pathophysiology and Transplantation, University of Milan, Milan, Italy

**Keywords:** Acute hypoxemic respiratory failure, Non-invasive respiratory support, High flow nasal cannula, Digital twins

## Abstract

**Background:**

Inconsistent responses to increased flow rates have been observed in patients with acute hypoxemic respiratory failure (AHRF) treated with high-flow nasal cannula (HFNC) therapy, with a significant minority in two recent studies exhibiting increased respiratory effort at higher flow rates. Digital twins of patients receiving HFNC could help understand the physiological basis for differing responses.

**Methods:**

Patient data were collated from previous studies in AHRF patients who were continuously monitored with electrical impedance tomography and oesophageal manometry and received HFNC at flow rates of 30, 40 or 45 L/min. Patients, based on their responses to an increase in flow rate to 60 L/min, were categorised into two groups: five responders with reduced oesophageal pressure swings ΔP_es_ (− 3.1 cmH_2_O on average), and five non-responders with increased ΔP_es_ (+ 2.0 cmH_2_O on average). Two cohorts of digital twins were created based on these data using a multi-compartmental mechanistic cardiopulmonary simulator. Digital twins’ responses to increased HFNC flow rates (60 L/min) were simulated with constant respiratory effort to assess changes in gas exchange and lung mechanics, and with varying respiratory effort to quantify their combined effects on lung mechanics and P-SILI indicators.

**Results:**

The digital twins accurately replicated patient-specific responses at all flow rates. Responder digital twins showed a mean 20 mL/cmH_2_O increase in lung compliance at higher flow rates, versus a 6 mL/cmH_2_O decrease in compliance with non-responders. In digital twins of responders versus non-responders, increased flow rates produced a mean change in lung stress of − 1.5 versus + 1.2 cmH_2_O, in dynamic lung strain of − 8.8 versus + 16.4%, in driving pressure of − 1.3 versus + 1.1 cmH_2_O, and in mechanical power of − 0.8 versus + 1.2 J/min. Higher flow rate dependent positive end-expiratory pressure in digital twins of non-responders did not cause recruitment, and reduced tidal volumes due to higher functional residual capacities—to compensate for the resulting worsened gas-exchange, non-responders increased their respiratory effort, in turn increasing patient self-inflicted lung injury (*P*-SILI) indicators. In digital twins of responders, reductions in tidal volumes due to higher FRCs resulting from increased PEEP were outweighed by alveolar recruitment. This increased compliance and improved gas exchange, permitting reduced respiratory effort and decreases in *P*-SILI indicators.

**Conclusions:**

Failure to reduce spontaneous respiratory efforts in response to increased HFNC flow rates could be due to a deterioration in lung mechanics, with an attendant risk of *P*-SILI.

**Supplementary Information:**

The online version contains supplementary material available at 10.1186/s40635-025-00773-5.

## Introduction

High-flow nasal cannula (HFNC) therapy is a form of non-invasive respiratory support commonly used as a first-line intervention for acute hypoxemic respiratory failure (AHRF) patients [1, 2]. HFNC delivers heated and humidified air, or oxygen, at flow rates exceeding 20 L/min [3], and when successful has been shown to improve oxygenation, lower respiratory effort, and reduce the need for mechanical ventilation [4]. Key mechanisms underlying the benefits of HFNC include enhanced CO_2_ clearance from the anatomical dead space and a flow rate-dependent positive airway pressure, which promotes alveolar recruitment.

Despite its benefits, how to choose the optimal flow setting for an individual patient during HFNC therapy remains uncertain, and studies reporting physiological responses to changes in flow rates (our focus here) are comparatively rare in the literature. Studies indicate that oxygenation generally improves with higher HFNC flow rates [5], but individual responses to increased flow rates can vary significantly. For example, some patients show no significant change or even a decreased ROX index with increased flow rates. Zhang et al. [6] observed no significant changes in the ROX index at various HFNC flow rates in postextubation patients with mild hypoxemia. Mauri et al. [7] found that 30% (17/57) of AHRF patients experienced an average 9% decrease in their ROX index when HFNC flow rates were increased from 30 to 60 L/min, while in another study [8] involving seventeen AHRF patients, 43% of patients had increased oesophageal pressure swings (ΔP_es_) when the flow rate was increased from 30 or 45 to 60 L/min.

These findings highlight the importance of further research into personalised adjustment of HFNC flow rates based on individual physiological responses to optimise patient outcomes and minimise the risk of lung injury. However, in vivo experiments to address this question face many technical, ethical, and practical challenges. Mechanistic digital twins that replicate the physiological and mechanical behaviour of individual patients using patient-specific data could allow for cost-effective and tightly controlled investigations of different clinical scenarios that are impractical to perform directly in patients. This study aims to use mechanistic digital twins, constructed based on data from previous clinical studies, to investigate the physiological basis behind the varying effects of increased HFNC flow rates on different patients.

## Methods

This section summarises the patient data extracted from measurements made in two cohorts of AHRF patients and outlines how these data were used to develop the digital twins employed in this study. Full methodological details are provided in the online supplement.

*Study population:* Anonymized individual patient data were obtained from two previous prospective randomised crossover studies [8, 9] conducted in non-intubated AHRF patients admitted to the ICU of Fondazione IRCCS Ca’ Granda Ospedale Maggiore Policlinico, Milan, Italy. These studies were selected, because the patients were monitored in an unusual level of detail (in both studies, patients were continuously monitored using oesophageal manometry and electrical impedance tomography (EIT)) and because of the range of responses to higher HFNC flow rates recorded:

- **Inclusion criteria**: Age > 18 years; presence of AHRF with a PaO_2_/FiO_2_ ratio < 300 mmHg.
- **Study 1 (2017)**: Included 17 patients, each undergoing four study phases in a computer-generated random order: standard non-occlusive oxygen facial mask (flow rate: 12 L/min) and HFNC therapy (flow rates: 30, 45, and 60 L/min), with each phase lasting 20 min [8]
- **Study 2 (2023)**: Included 10 patients, each receiving support with an asymmetrical interface and a conventional (symmetrical) interface in randomized order, at flow rates of 40 and 60 L/min [9]

Selected patients from these two studies were categorised into two groups (responders and non-responders) based on their physiological responses to increased HFNC flow rates:

- **Responders (N = 5)**: Patients whose Δ*P*_es_ decreased by at least 0.5 cmH_2_O in response to flow rate increasing to 60 L/min
- **Non-responders (N = 5)**: Patients whose Δ*P*_es_ increased by at least 0.5 cmH_2_O in response to flow rate increasing to 60 L/min.

Detailed individual patient data are shown in Table 1. Tidal volume was measured using an electrical impedance tomography (EIT) system comprising a dedicated belt with 16 evenly spaced electrodes positioned around each patient’s thorax at the fifth or sixth intercostal space. This belt was connected to a commercial EIT monitor (PulmoVista^®^ 500; Dräger Medical GmbH, Lübeck, Germany). During the study, the EIT system applied alternating electrical currents at 20 Hz around the thorax, enabling tomographic image acquisition every 50 ms. All data were stored for offline analysis using specialised software (Dräger EIT Data Analysis Tool and EITdiag). Before initiating the study protocol, spirometry was recorded during 30 s of spontaneous breathing using a mouthpiece with nasal occlusion. This served to calibrate the EIT data offline. Specifically, 3–5 representative tidal volumes were selected from the spirometry and matched with corresponding EIT impedance changes. The average ratio between millilitres and arbitrary impedance units was then used to convert impedance variations into volume changes throughout the study. Following calibration, the mouthpiece and spirometer were removed, and patients resumed unimpeded breathing. Respiratory effort was measured with oesophageal pressure monitoring to estimate tidal swings in pleural pressure, and by manual recording of respiratory rate [8, 9].

**Table 1:** Detailed data of responder and non-responder patients

		HFNC flow rate (L/min)	FiO_2_ (%)	RR (bpm)	PaO_2_ (mmHg)	PaCO_2_ (mmHg)	VT (mL)	ΔP_es_ (cmH_2_O)
Non-responder	1	30	40	18	94.0	46.0	113.5	3.7
		60	40	20	116.0	44.0	168.7	5.03
	2	45	45	22	88.0	47.0	401.68	4.45
		60	45	21	94.0	50.0	379.63	5.17
	3	40	41	20	93.0	32.0	619.0	12.22
		60	44	22	111.0	30.0	631.0	13.01
	4	40	40	33	84.0	44.0	309.36	10.74
		60	40	35	100.0	40.0	275.52	13.64
	5	40	30	24	89.0	34.0	729.6	8.03
		60	30	23	94.0	34.0	753.4	12.17
Responder	1	30	50	32	80.9	36.6	152.26	15.74
		60	50	31	97.4	38.2	137.34	9.06
	2	30	70	14	69.0	34.0	558.97	15.78
		60	70	14	97.0	34.0	500.51	14.32
	3	45	70	10	101.0	36.0	435.74	11.99
		60	70	10	108.0	36.0	492.18	10.09
	4	45	70	18	102.0	47.5	319.29	8.31
		60	70	19	104.0	42.3	347.12	6.19
	5	40	30	20	86.0	35.0	583.0	8.39
		60	30	16	92.0	35.0	575.0	5.05

*HFNC simulator:* This study utilises a validated multi-compartmental computational simulator of the cardiopulmonary system, which has previously been employed to simulate patients with COVID-19 [10], chronic obstructive pulmonary disease (COPD) [11], acute respiratory distress syndrome (ARDS) [12, 13], and AHRF [14]. Key advantages of this model include the ability to define multiple alveolar compartments with unique mechanical properties, such as alveolar collapse, consolidation, stiffening, gas exchange disruption, and airway obstruction. This enables the simulation of clinical features, such as ventilation–perfusion mismatch, physiological shunt, gas trapping with intrinsic positive end-expiratory pressure (PEEP), alveolar reopening, etc. [15, 16]. The version of the simulator used here incorporates key mechanisms underlying HFNC therapy, including carbon dioxide clearance from dead space, gas leakage, turbulent flow resistance, increases in airway resistance at higher flow rates, and a flow rate dependent PEEP [17]. A detailed description of the simulator can be found in the online supplement.

*Creation of digital twins:* Digital twins were constructed using the individual patient data from [8, 9]. Inputs to the simulator included gender, age, height, weight, fraction of inspired oxygen (FiO_2_), and HFNC flow rate. The residual volume and chest wall compliance were estimated using standard equations in the literature [18–20]. A Genetic algorithm (GA) encoded in the MATLAB optimisation toolbox was used to calibrate the simulator’s parameters so that each digital twin replicates the patient’s response to HFNC therapy, in terms of arterial blood gases (PaO_2_ and PaCO_2_), Δ*P*_es_, and tidal volume (VT). An optimisation problem for model calibration was formulated to minimise the difference between the model predictions and the actual measurements from the patient. The model parameters optimised during this process include the extrinsic pressure (*P*_ext,i_) and stiffness coefficient $\left( {k_{i} } \right)$ for each alveolar compartment, as well as values for Respiratory Quotient (RQ), Oxygen Consumption factor (VO_2,factor_), haemoglobin concentration (Hb), Base Excess (BE), Cardiac Output factor (CO_factor_), resistance of the bronchi and bronchioles (*R*_*B*_), maximum muscle pressure (*P*_mus_), and duty cycle (DC). Table S5 in the online supplement shows the allowed range of variation for these model parameters during the optimisation.

Model calibration was performed at two flow rates simultaneously: a baseline HFNC flow rate (30, 40, or 45 L/min) and a higher flow rate (60 L/min). Model parameters defining the patient’s respiratory pathophysiology were kept constant at both flow rates, while the patient’s muscle pressure (*P*_mus_) was allowed to vary to reflect changes in their respiratory effort recorded at different flow rates. The cost function $\left( J \right)$, representing the error to be minimised by the optimisation algorithm is defined as

$$ \mathop {\min }\limits_{x} J = \sqrt {\mathop \sum \limits_{i = 1}^{4} w_{i} \left( {\frac{{\hat{Y}_{{i@{\text{base flow rate}}}} - Y_{{i@{\text{base flow rate}}}} }}{{Y_{{i@{\text{base flow rate}}}} }}} \right)^{2} } + \sqrt {\mathop \sum \limits_{i = 1}^{4} w_{i} \left( {\frac{{\hat{Y}_{i@60L/\min } - Y_{i@60L/\min } }}{{Y_{i@60L/\min } }}} \right)^{2} } $$

where $Y = \left[ {{\text{PaO}}_{2, } {\text{PaCO}}_{2, } \Delta {\text{Pes}},{\text{VT}}} \right]$ are measured patient data, $\hat{Y}$ are the corresponding model outputs, and $w_{i}$ are weighting functions chosen to balance the accuracy of the matching among the different outputs (see Table S6). The optimisation algorithm was terminated when either the cost function tolerance (change in the best fitness value from one generation to the next) fell below 10⁻^4^ or 250 generations were reached.

To increase the generalizability of our results, an additional sensitivity analysis was conducted by creating 10 additional digital twins from each original by allowing the model parameters determining its lung characteristics (*P*_ext,i_, $k_{i}$, RQ, VO_2,factor_, Hb, BE, CO_factor_, *R*_*B*_, and *P*_mus_) to vary by $\pm { }10{\text{\% }}$ and randomly sampling from a uniform distribution.

*Simulating the effects of increased flow rate:* The patient’s response to a different flow rate, in terms of their respiratory effort, comprises two parts (1) the change in the force/pressure applied to their respiratory muscles (*P*_mus_), and (2) the frequency of their breathing. The former could not be measured in vivo but was estimated in the digital twins during model matching based on the available data on Δ*P*_es_ and VT, while the latter is reported in the data as respiratory rate. Consequently, following model calibration, digital twins’ responses to an increased HFNC flow rate (60 L/min) were recorded in two scenarios:

- While maintaining respiratory effort constant at the levels observed at the baseline flow rate; this step aimed to uncover the changes in gas-exchange and lung mechanics due to higher flow rates that, via neural feedback, could have produced the differing changes in patients’ spontaneous respiratory effort observed after the HFNC flow rate was increased.
- While varying patients’ respiratory effort according to the data on changes in RR and Δ*P*_es_ at higher flow rates; this step aimed to quantify the combined effects of higher flow rates and changes in patients’ spontaneous respiratory effort on lung mechanics and P-SILI indicators.

These steps aimed to uncover the changes in gas-exchange that, via neural feedback, could have produced the differing changes in patients’ spontaneous respiratory effort observed after HFNC flow rate was increased.

*Statistical analysis:* Statistical analysis was not performed owing to the purely deterministic and mechanistic nature of the simulations, i.e., changes in digital twin outputs were always only due to corresponding changes in identifiable model parameters, rather than due to random chance, as could potentially be the case in in vivo studies.

## Results

**Fig. 1:**
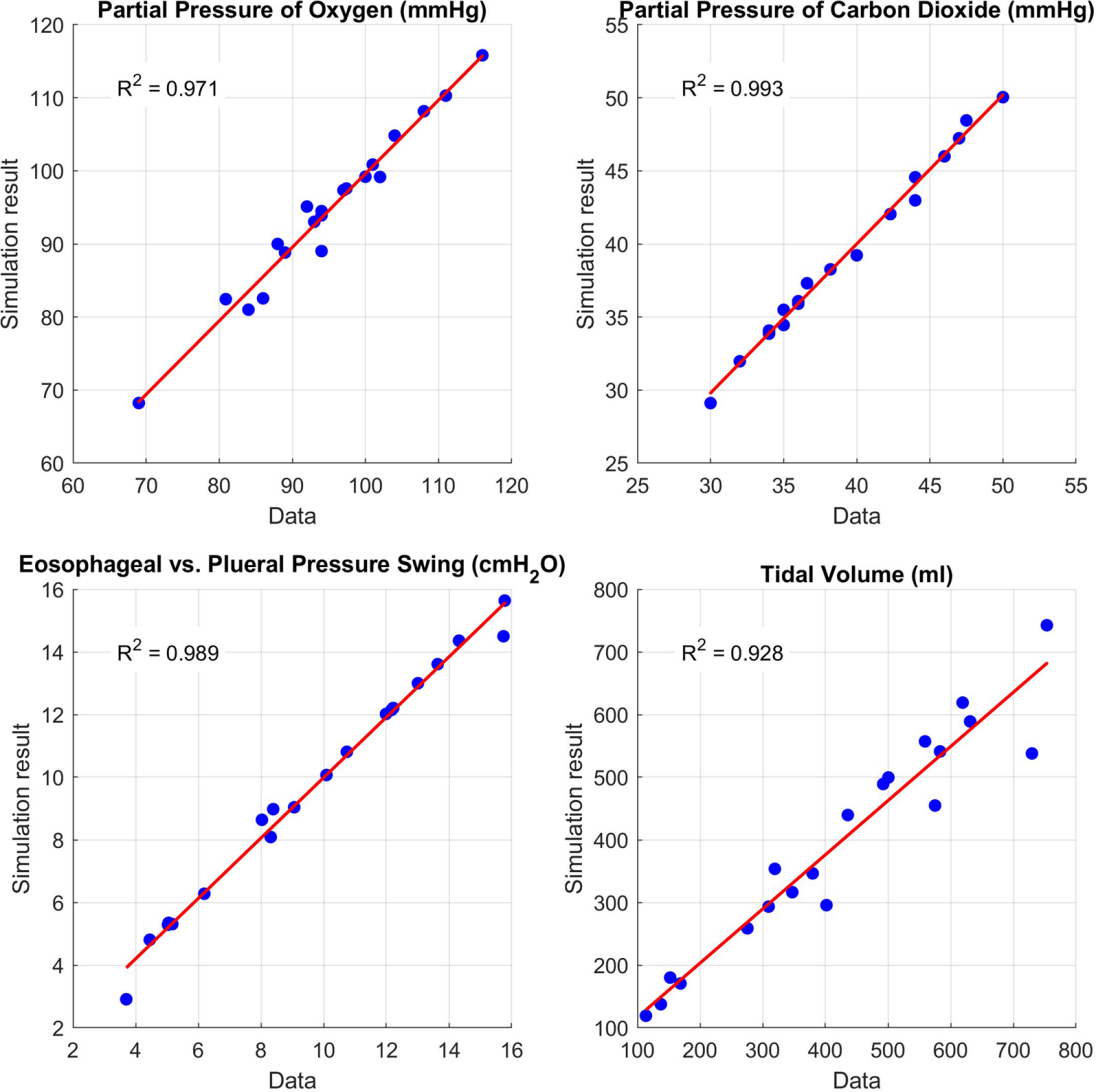
Patient data compared to simulator outputs

*Digital twins accurately reproduce patient measurements:* The digital twins effectively replicated all measured parameters from the original patient cohort. Comparisons of digital twin outputs with patient measurements for PaO_2_, PaCO_2_, pleural pressure swing (Δ*P*_pl_), and VT are shown in Fig. 1. For Δ*P*_pl_, the actual pleural pressure change from the simulator was compared to its surrogate, Δ*P*_es_ (measured via oesophageal manometry). The mean absolute percentage error and mean absolute bias between digital twins and patient data were as follows: 1.42%/1.29 mmHg for PaO_2_, 0.91%/0.35 mmHg for PaCO_2_, 3.64%/0.25 cmH_2_O for ΔP_pl_, and 7.76%/34.45 mL for VT.

**Fig. 2:**
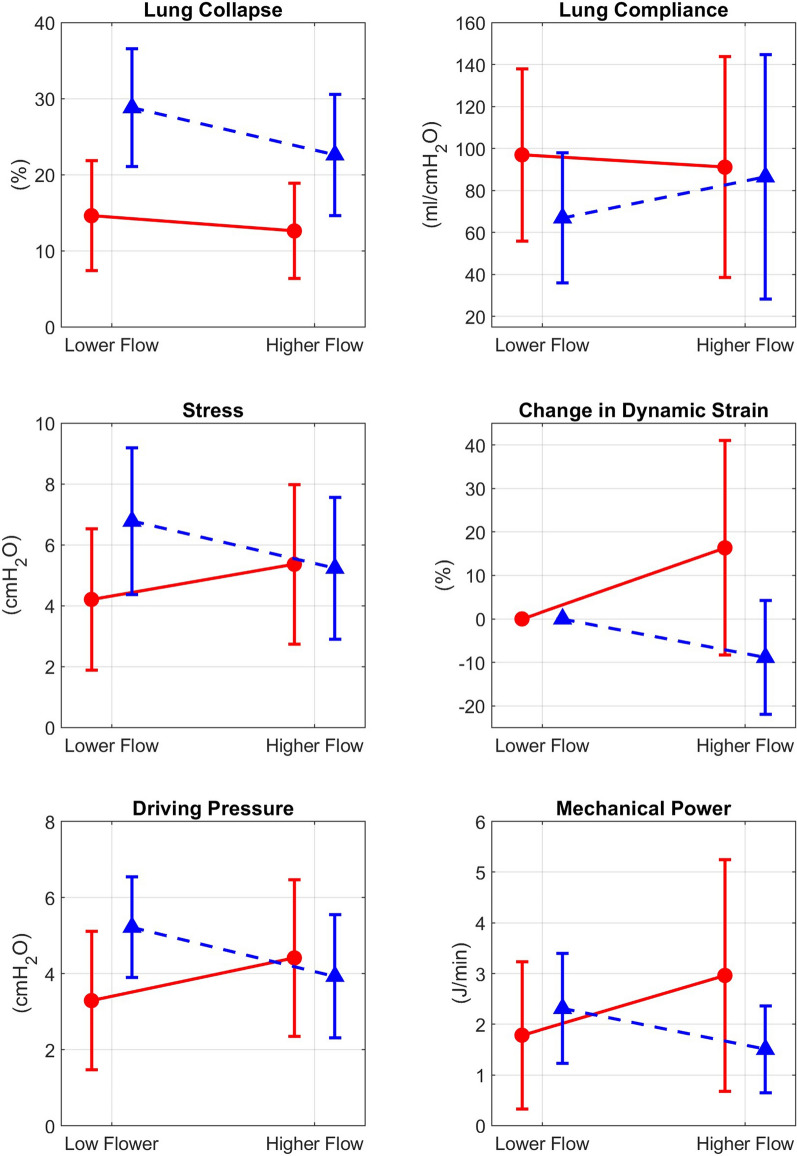
Mean (standard deviation) of lung mechanics parameters and *P*-SILI indicators in responders (blue dashed line) and non-responders (red solid line)

*Lung mechanics parameters and P-SILI indicators in Responders and Non-responders:* Fig. 2 shows the percentage of lung collapse, lung compliance, total lung stress, percentage change in lung dynamic strain, driving pressure, and mechanical power, calculated in the digital twins of the responders and non-responders. Responders demonstrated significant benefits at higher HFNC flow rates compared to non-responders: lung collapse decreased by 6.2% in responders versus 2% in non-responders, Lung compliance increased by 20 mL/cmH_2_O in responders but decreased by 6 mL/cmH_2_O in non-responders.

Patient Self Inflicted Lung Injury (*P*-SILI) indicators decreased in responders while increasing in non-responders. The change in mean total stress was − 1.5 versus + 1.2 cmH_2_O, the change in mean dynamic lung strain was − 8.8 versus + 16.4%, the change in driving pressure was − 1.3 versus + 1.1 cmH_2_O, and the change in mechanical power was − 0.8 versus + 1.2 J/min, for the responders versus non-responders.

Digital twins of non-responders had higher dead space-to-shunt ratio, particularly at higher flow rates, compared to responders (see Table S7 in the SM).

**Fig. 3:**
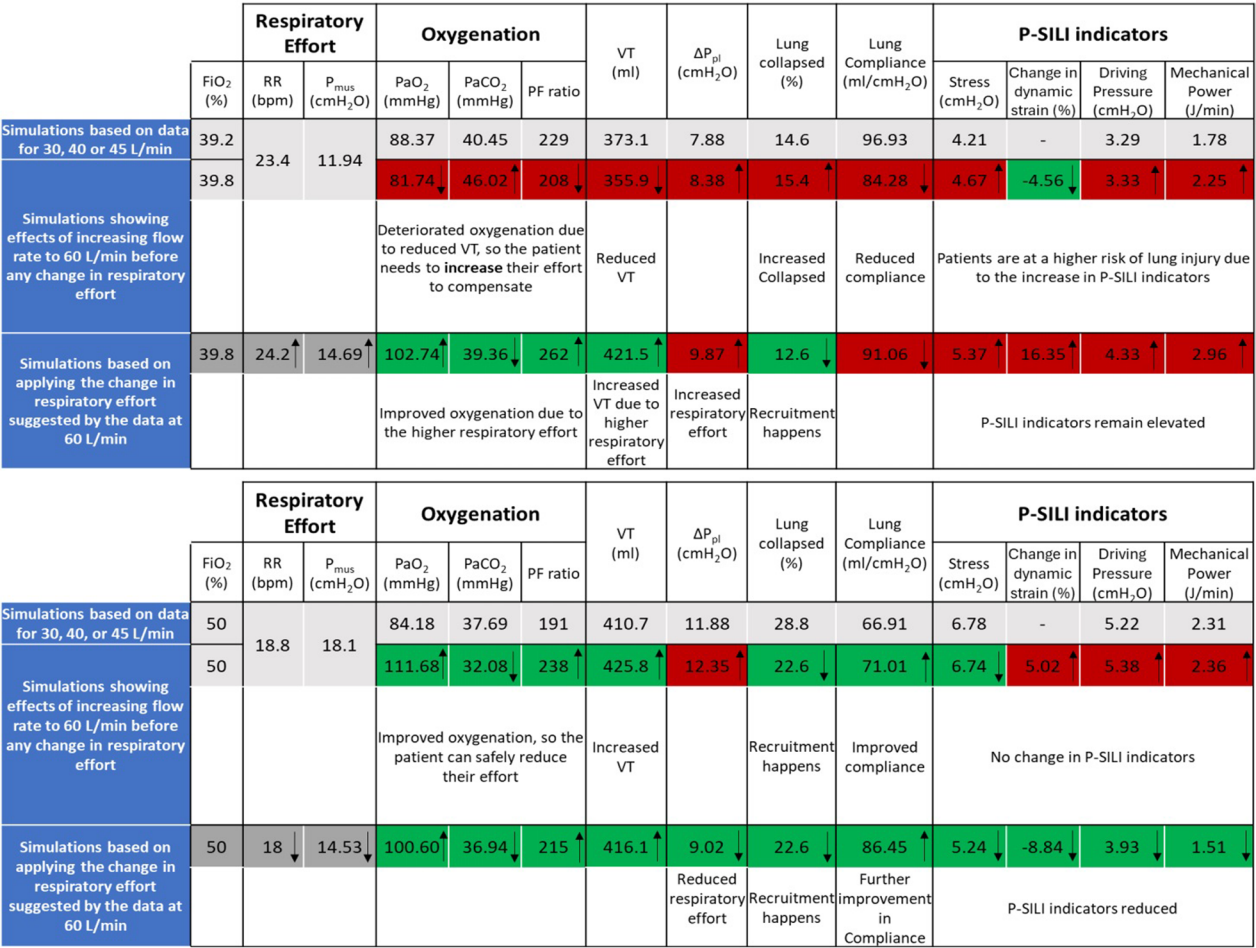
Digital twin analysis of non-responders and responders, average values at different flow rates and different levels of spontaneous respiratory effort

*Effect of increasing flow rate to 60 L/min with constant respiratory effort:* Fig. 3 shows the responses of the digital twins when the HFNC flow rate was increased to 60 L/min, while respiratory effort was kept constant. In responders, lung recruitment improved (6.2% fewer collapsed alveoli on average), leading to a mean 4.1 mL/cmH_2_O increase in lung compliance and enhanced oxygenation without changes in P-SILI indicators. In non-responders, lung collapse increased by 0.8% on average, resulting in a mean 12.65 mL/cmH_2_O decrease in lung compliance and worsened oxygenation. P-SILI indicators also increased under these conditions.

*Sensitivity analysis:* To increase the generalizability of our results and ensure that they do not depend on a unique model parameterisation, we recomputed all the above results in two cohorts of 55 digital twins, generated from the originals using randomised sampling of key model parameters. Results are presented in Figures S15 and S16 of the online supplement and are consistent with all results presented above.

## Discussion

HFNC settings significantly influence clinical outcomes in AHRF patients [21]. Previous studies have demonstrated that increasing flow rates improves oxygenation [5, 22] and lung compliance while reducing the work of breathing (WOB) [23]. These effects are the results of enhanced carbon dioxide clearance [24, 25], recruitment of collapsed alveoli, and increased end-expiratory lung volume and PEEP [26, 27]. However, the level of heterogeneity in patient responses observed in a number of previous studies underscores the need for personalised flow titration to balance benefits against risks, such as alveolar overdistension [28–30] and patient discomfort [31, 32].

This study demonstrates the potential of mechanistic digital twins to help elucidate the patient-specific physiology underlying responses to HFNC therapy in AHRF patients. By replicating individual patient data and enabling completely controlled virtual experiments, digital twins can provide valuable insights into the factors driving variability in clinical outcomes.

The results highlight potentially distinct patterns in the digital twins of responders and non-responders to increased HFNC flow rates. In the digital twins, responders benefited from lung recruitment at higher flow rates, which increased VT and compliance, leading to improved oxygenation. This allowed reductions in their respiratory effort without compromising recruitment, thereby maintaining adequate gas exchange, further improving compliance, and decreasing *P*-SILI indicators. Conversely, digital twins of non-responders experienced no recruitment, while reduced VT and compliance resulted in impaired gas exchange. To compensate, non-responders increased their respiratory effort, which partially restored VT, improved compliance, and enhanced oxygenation, but may also have resulted in increases in all P-SILI indicators (Fig. 3). An additional analysis in non-responders showed that these individuals reported higher dead space-to-shunt ratio, particularly at higher flow rates, compared to responders (Table S7 in the SM). Elevated dead space dampens the efficacy of PEEP in improving ventilation–perfusion (V/Q) matching [33], as perfusion in ventilated regions is already compromised, reducing the potential for effective alveolar recruitment [34]. At higher flow rates, this imbalance can lead to overdistension in preserved lung regions, compressing capillaries, exacerbating V/Q mismatch, and redistributing blood flow to poorly ventilated or non-ventilated zones, thus resulting in worsened gas exchange [35, 36]. Furthermore, these changes also drive compensatory increases in respiratory effort and ventilatory drive to maintain effective gas exchange [37]. Thus, the heightened work of breathing observed in non-responders could reflect the physiological burden imposed by elevated dead space [38].

Taken together, these findings underscore the need for personalised HFNC titration strategies to avoid potential harm while maximising therapeutic benefits while also highlighting the crucial importance of close monitoring of changes in patients’ spontaneous respiratory efforts after adjusting flow rates. HFNC delivers a flow rate dependent level of PEEP to the patient—interestingly, our findings are highly consistent with those of a recent in vivo study investigating the effects of high versus low PEEP in patients with ARDS exhibiting intense inspiratory effort during non-invasive ventilation [39].

This study has some limitations, the most important being that the digital twins were constructed using data from a limited sample size—additional studies using larger patient data sets would be necessary to confirm our results and allow them to be generalised to broader patient populations. In addition, while the digital twins accurately replicate the measured patient parameters, not all relevant patient parameters were (or can be) measured, a limitation which could affect our results. Finally, certain clinically relevant aspects of HFNC therapy, such as patient comfort and interface variability, were not directly assessed.

## Conclusions

This study demonstrates the potential of digital twins for investigating the mechanisms underlying the differing physiological responses to higher HFNC flow rates that have been observed in recent studies. Higher flow rates generally improve gas-exchange and reduce inspiratory effort, but the effects are highly heterogeneous. Our analysis suggests that while responders may benefit from alveolar recruitment and reduced *P*-SILI indices, non-responders could face risks of alveolar overdistension and worsened *P*-SILI indices. Further research is warranted to refine and expand these research tools using additional patient data and to evaluate their broader clinical applicability in real-world settings.

## Supplementary Information

Additional file 1.

## Data Availability

All data generated or analysed during this study are included in this published article and its supplementary information file.

## Abbreviations

HFNC: High Flow Nasal Cannula
AHRF: Acute Hypoxemic Respiratory Failure
*P*-SILI: Patient-Self Inflicted Lung Injury
EELI: End Expiratory Lung Impedance
ICU: Intensive Care Unit
EIT: Electrical Impedance Tomography
PEEP: Positive End Expiratory Pressure
COPD: Chronic Obstructive Pulmonary Disease
ARDS: Acute Respiratory Distress Syndrome
ΔP_es_: Oesophageal pressure swing
PaO_2_: Partial Pressure of Arterial Oxygen
PaCO_2_: Partial Pressure of Arterial Carbon Dioxide
ΔP_pl_: Tidal Change in Pleural Pressure
VT: Tidal Volume
NIV: Non-Invasive Ventilation
*P*-SILI: Patient-Self Inflicted Lung Injury
EELI: End Expiratory Lung Impedance
FiO_2_: Fraction of Inspired Oxygen
RR: Respiratory Rate
GA: Genetics Algorithm
P_mus_: Maximum Muscle Pressure
